# Analysis of Influencing Factors Related to Health Literacy of Diabetic Patients: A Survey Based on DHLEIS

**DOI:** 10.1155/jdr/5110867

**Published:** 2024-11-25

**Authors:** Yalan Chen, Zepeng Wang, Fangyuan Jiang, Junyi Shi, Kui Jiang

**Affiliations:** Department of Medical Informatics, School of Medicine, Nantong University, Nantong, Jiangsu, China

**Keywords:** diabetes, evaluation index system, health literacy, influencing factors

## Abstract

**Purpose:** This study is aimed at investigating health literacy (HL) among diabetes mellitus (DM) patients using a comprehensive, scientific, feasible, and suitable HL assessment indicator system tailored for the diabetic population in mainland China and systematically analyzing the factors influencing HL in this population.

**Methods:** The Delphi expert consultation method was employed to initially draft and refine the Diabetes Health Literacy Evaluation Indicator System (DHLEIS). The reliability and representativeness of the indicator system were tested through metrics including the active coefficient, authority degree, and coordination degree. A HL survey questionnaire for diabetic patients was developed based on DHLEIS and administered to diabetic patients across five hospitals in Nantong and Yancheng cities, Jiangsu Province. The random forest method was utilized to deeply analyze the impact of various factors on HL and its four dimensions and to identify the core influencing factors.

**Results:** Analysis of 707 questionnaires based on the DHLEIS revealed that nine factors—age, sex, body shape, income, exercise, education level, duration of DM, whether insulin is injected, and the number of cohabitants—significantly impact the HL levels. Among these, age, duration of DM, education level, and number of cohabitants were particularly influential across the four dimensions of health knowledge, awareness, behavior, and skills. Factors related to health knowledge and skills were the most significant contributors to overall HL.

**Conclusions:** The multidimensional analysis of factors influencing HL offers valuable insights into characterizing varying levels of HL among diabetic patients. This approach supports targeted cognitive improvements and the effective enhancement of health skills, ultimately leading to better health outcomes.

## 1. Introduction

Diabetes mellitus (DM) is a chronic noncommunicable disease caused by metabolic disorders influenced by factors such as genetics and environment. These disorders can result in chronic damage, dysfunction, or even failure of multiple organs. With its high prevalence, disability rate, and mortality rate, DM poses a silent but serious threat to global health. It is estimated that the number of diabetic patients will rise to 643 million by 2030 and 783 million by 2045 [1, 2]. China, which has the largest population of individuals with DM, faces significant challenges in its prevention and management. The rapid pace of social and economic development, lifestyle changes, accelerated urbanization, an aging population, and other factors have all contributed to a marked increase in the prevalence of DM in China.

In the 1970s, Simonds first introduced the concept of “health literacy” (HL) at a health education conference, advocating for the establishment of minimum standards for HL [3]. Over the past half-century, the definition and scope of HL have been continuously refined and expanded. The most widely recognized definition describes HL as the ability of individuals to obtain, understand, and use basic health information and services to make informed health decisions, thereby maintaining and promoting their health [4]. For diabetic patients, HL specifically refers to their ability to gather, comprehend, and use health education information and healthcare services related to DM, with the goal of managing and improving their condition [5].

Research indicates that over 80% of chronic diseases can be prevented by adopting a healthy lifestyle [6]. In 2022, the American Diabetes Association and the European Association for the Study of DM released a consensus report recommending that social determinants of health, healthy lifestyle behaviors, and DM self-management education and support be considered integral components of DM care. However, achieving these goals requires patients to have good HL, which is crucial for managing their health and making appropriate health decisions [7]. Studies have shown that diabetic patients with inadequate HL often lack essential health-related knowledge, demonstrate poorer medication adherence, have weaker communication and interaction with healthcare professionals, and participate less frequently in health decision-making [8]. Therefore, systematically studying the factors that influence HL in diabetic patients and effectively improving their HL levels are vital strategies for the prevention and control of DM.

Although most studies suggest that there is still a lack of a universally recognized screening tool to assess HL [9], the rapid development of artificial intelligence (AI) technology and the growing emphasis on electronic health literacy (EHL) have introduced new dimensions to research on HL assessment and influencing factors. Brown et al. [10] explored the use of natural language processing techniques and machine learning (ML) to develop a novel, effective, and scalable method for measuring patient HL. Similarly, Hæsum, Cichosz, and Hejlesen [11] applied ML methods to refine the influencing factors and evaluation items of HL in patients with chronic obstructive pulmonary disease, aiming to create a concise HL assessment tool. To date, AI has seen extensive applications in DM risk prediction [12], diabetic retinopathy detection [13], and wearable device-assisted intelligent DM management [14, 15]. In the fields of HL and health education, emerging research includes the use of ML to identify key sociodemographic variables for HL modeling [16] and the development of AI-based precise linkage systems for health education [17].

A systematic evaluation of HL intervention trials for diabetic patients in mainland China revealed numerous influencing factors, a wide range of intervention models, and various assessment scales that are often overly broad and lack comprehensive evaluation dimensions [18, 19]. This highlights the need for a scientific, comprehensive, and tailored HL evaluation indicator system specifically designed for the diabetic population in mainland China. Such a system would enable accurate assessment of the core factors influencing HL among diabetic patients and effectively enhance their HL levels. To achieve this, the study employed the Delphi expert consultation method to develop the Diabetes Health Literacy Evaluation Indicator System (DHLEIS) and designed a multidimensional questionnaire to investigate the factors affecting HL in diabetic patients. This study is aimed at providing a theoretical foundation for medical professionals to quickly assess the HL of diabetic patients and more precisely address adverse factors.

## 2. Materials and Methods

### 2.1. Determination of the Indicator System and Weights

#### 2.1.1. Construction of the DHLEIS Framework

Based on the concepts and definitions of HL and EHL and incorporating the knowledge–attitude–practice theory as well as the “66 Health Literacy Principles” [20], a preliminary two-tier indicator system framework for assessing the HL of diabetic patients was developed. Experts rated each indicator on a 5-point scale: *very important* (5 points), *quite important* (4 points), *average* (3 points), *not very important* (2 points), and *not important* (1 point).

#### 2.1.2. Weight determination

After two rounds of expert consultations and feedback, the indicator system was refined, and the weights of each level of indicator were determined using the coefficient of variation method.

### 2.2. Development of the HL Questionnaire

A HL questionnaire for diabetic patients was designed based on the DHLEIS framework. After multiple rounds of pretesting and revisions, the final version of the HL survey for diabetic patients was established. The reliability and validity of the questionnaire were assessed using Cronbach's *α* coefficient, coordination coefficient, and authority coefficient.

### 2.3. Study Population

According to the basic principles of questionnaire development and psychometric properties, there is a positive relationship between the number of items on a scale and the required sample size, with a ratio of five to 10 participants per item [21]. Given that the questionnaire consists of 39 items, the required sample size ranges from 195 to 390 participants. The inclusion criteria for participants were adults aged 21 and over who met the World Health Organization (WHO) diagnostic standards for DM and were capable of independently communicating with the interviewers. Exclusion criteria included pregnant women and individuals with cognitive impairments.

Following a preliminary study, an anonymous survey was conducted using convenience sampling from January 2022 to December 2022 in the outpatient and inpatient departments of five hospitals in Nantong and Yancheng cities, Jiangsu Province. A total of 820 diabetic patients voluntarily participated and completed the survey. Prior to the survey, interviewers were recruited and trained at each of the five hospitals to ensure consistent data collection.

### 2.4. ML and Visualization Analysis

Random forest (RF) is a ML ensemble algorithm used for classification and regression, capable of effectively identifying key influencing factors by integrating multiple decision trees [22]. Given the diversity and complex nonlinear relationships among HL-related variables, RF contribution analysis was employed to extract the core factors influencing HL levels in diabetic patients. Principal component analysis (PCA) is commonly used to assess the dimensional structure of datasets or to reduce a large number of variables into a smaller set of linear combinations, facilitating hierarchical clustering or association determination [23, 24]. In this study, PCA was used to validate the effectiveness of the ML approach. Statistical analyses of the relevant influencing factors were conducted using RStudio, while multidimensional visualization analyses were performed using OriginPro software.

## 3. Results

### 3.1. Questionnaire Design Based on the DHLEIS Framework

After two rounds of expert consultations and feedback revisions, the DHLEIS framework and content were finalized, comprising four primary indicators (health awareness, health knowledge, health behavior, and health skills) and 39 secondary indicators. The specific content and weights of each indicator are presented in Table 1.

A corresponding survey questionnaire was developed based on the DHLEIS (detailed content is available in Supporting Information S1). The questionnaire categorizes the overall influencing factors of diabetic patients' HL into two sections: (1) personal basic information, including variables such as sex, age, duration of illness, and education level; and (2) information across the four dimensions, with each of the 39 secondary indicators corresponding to a specific question. The questionnaire includes two main types of questions: (1) multiple-choice questions that assess knowledge or skills, with scores assigned based on the number of correct answers, up to a maximum of 5 points; and (2) Likert 5-point scale questions, with scores ranging from 1 to 5 based on the selected response.

The survey questionnaire demonstrated strong internal consistency, with an overall Cronbach's *α* coefficient of 0.904 and coefficients ranging from 0.848 to 0.875 across the four dimensions. The authority coefficient of the DHLEIS expert consultation results was 0.89, indicating a high level of expert agreement. The concordance coefficients from the two rounds of expert consultations were 0.362 and 0.358, respectively, suggesting substantial expert approval of the content of the evaluation indicators. Therefore, the survey questionnaire is considered to meet validity requirements.

### 3.2. Basic Information of the Survey Subjects

A total of 820 questionnaires were distributed, and 800 were returned. After excluding those with a large number of missing values, 707 valid questionnaires remained for analysis. The basic information of the survey subjects is presented in Table 2.

Based on the HL scores, the study subjects were divided into two groups: the HL-deficient group, consisting of 320 cases (45.3%) with scores below 130 points, and the HL-adequate group, consisting of 387 cases. Univariate analysis revealed significant differences in HL scores among diabetic patients by sex, age, duration of disease, education level, average monthly income, exercise, and the number of cohabitants (*p* < 0.05).

### 3.3. The Impact of Basic Information-Related Factors on HL Level

#### 3.3.1. Analysis of the Importance of Basic Information on HL Level

**Figure 1:**
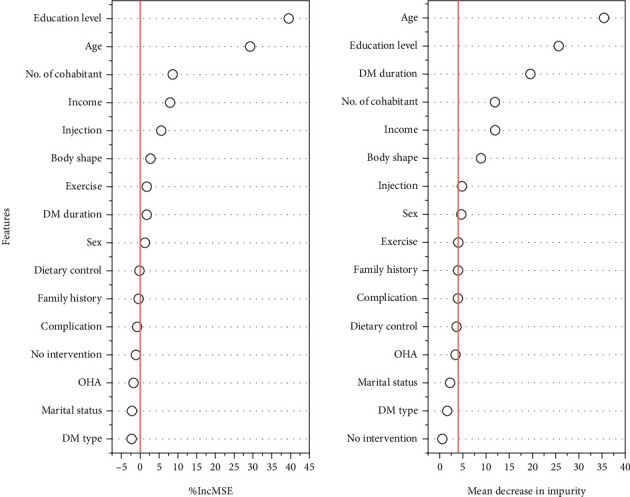
Analysis of the importance of basic information on health literacy. The further to the right a point is, the greater the impact of that factor on health literacy. DM, diabetes mellitus; OHA, oral hypoglycemia agent.

Using the %IncMSE and mean decrease in impurity (MDI) metrics from the RF analysis, the importance of basic information related to HL was ranked (Figure 1). The results indicate that nine factors—age, sex, body type, income, exercise, education level, DM duration, whether insulin is injected, and the number of cohabitants—are important in both metrics. Notably, age and education level had the most significant impact across both analyses.

#### 3.3.2. Density Analysis of HL Levels Across Different Demographic Characteristics (User Profiling)

**Figure 2:**
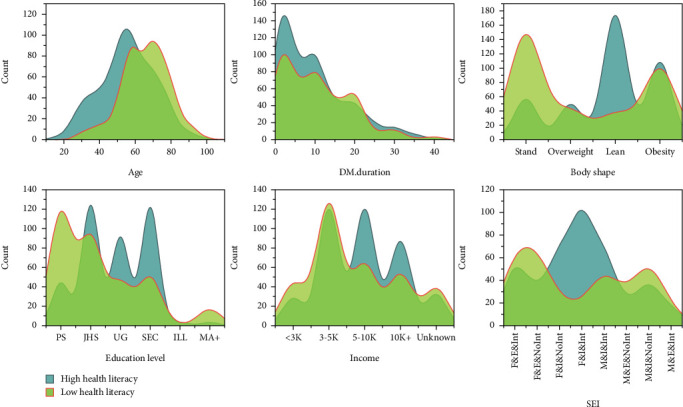
Distribution of health literacy levels across different demographic characteristics. Horizontal axis represents different characteristic values or categories. Vertical axis represents counts (population density). Color represents two levels of health literacy. DM, diabetes mellitus; F, female; M, male; Int, insulin injection; NoInt, no insulin injection.

Density plots of HL levels across key influencing factors were used to depict user profiles for different HL levels (Figure 2). The analysis shows significant differences in the density distribution of HL levels in relation to body type, marital status, income, and education level. The age density plot reveals that older populations tend to have a higher proportion of lower HL. In the distribution plot for education level, individuals with higher education levels generally exhibit higher HL. User profiling based on HL levels can provide theoretical support for the development of personalized health intervention measures.

### 3.4. The Impact of Basic Information Factors on the Four Dimensions of HL

#### 3.4.1. Analysis of the Contribution of the Four Dimensions of HL to the Overall HL Level

The impact and contribution of the four dimensions of HL—health awareness, health knowledge, health behavior, and health skills—on the overall HL level were assessed using RF and PCA (Table 3). The total HL score served as the outcome variable, while the scores from each dimension were used as input variables for constructing the RF model. The MDI metric reflects each variable's contribution to the model's classification performance, with the higher values indicating greater importance. PCA was used to understand the proportion of variance each dimension contributes to the overall model, serving as a comparative reference. The results indicate that the contributions identified by both methods are generally consistent, with health knowledge and health skills contributing more significantly to HL levels, while the impact of health awareness and health behavior is comparatively lower.

#### 3.4.2. Analysis of the Importance of Basic Information Factors on the Four Dimensions of HL

**Figure 3:**
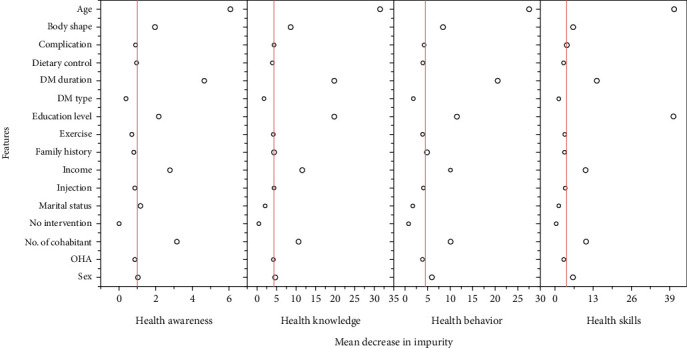
The impact of different influencing factors on the four dimensions of health literacy (health skills, health behavior, health knowledge, and health awareness). The further to the right a point is, the greater the impact of that factor on the respective health dimension. DM, diabetes mellitus; OHA, oral hypoglycemia agent.

To better understand the impact of basic information factors on the four dimensions of HL, MDI analysis was employed to evaluate the importance of various factors across these dimensions (Figure 3). The results indicate that age, DM duration, education level, and the number of cohabitants significantly influence all four dimensions of HL. However, the effect of individual factors varies across the dimensions. For instance, health awareness shows little variation between sexes, but notable differences exist in health skills, behaviors, and knowledge (*p* < 0.05). Particularly in health skills and knowledge, males exhibit a significant advantage, whereas females demonstrate higher implementation (health behavior).

#### 3.4.3. Correlation Analysis Between Basic Information Factors and Four Dimensions of HL

**Figure 4:**
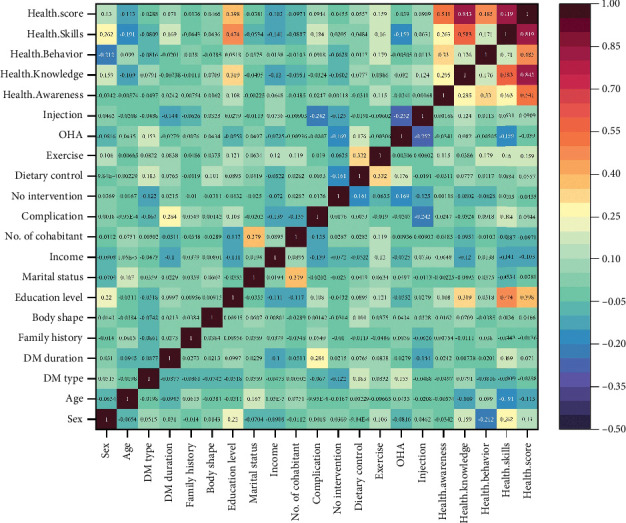
Correlation analysis of basic information factors on the four dimensions of health literacy. Changes in color and numerical values represent different levels of correlation, ranging from strong negative correlation (dark red, close to −1.0) to strong positive correlation (dark green, close to 1.0). DM, diabetes mellitus; OHA, oral hypoglycemia agent.

The correlation matrix between different factors of diabetic patients' HL (Figure 4) reveals a strong positive correlation between health knowledge and health skills (0.583), indicating that greater health knowledge can significantly enhance health skills. The correlations between education level and both health knowledge and health skills are also relatively strong (0.319 and 0.474, respectively), suggesting that higher education levels are associated with better health knowledge and skills. Conversely, the type of DM shows a negative correlation with both health awareness (−0.02) and health knowledge (−0.04). These findings provide insights for researchers and healthcare professionals into the factors influencing the health behavior and management of diabetic patients, aiding in the design of more effective interventions.

### 3.5. Layered Precision Analysis Based on the DHLEIS

**Figure 5:**
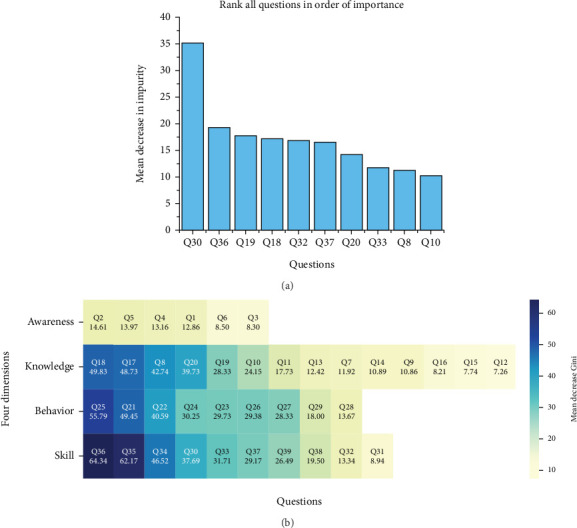
Impact of secondary indicator system factors on overall and dimensional health literacy. (a) Screening of core secondary indicator factors for health literacy in diabetic patients. (b) Importance ranking of secondary indicator factors for each health dimension (the darker the color, the higher the importance).

This section conducts a layered precision analysis of the key secondary indicators and direct factors affecting the HL levels of diabetic patients based on the DHLEIS system. Through the RF model, core secondary indicator factors influencing the HL of diabetic patients were identified (Figure 5(a)). The results indicate that Q30 (corresponding to D1: can read written information such as medical orders and drug instructions) has a significant impact on overall HL. The heat map of the importance of key questions for each dimension (primary indicators) in Figure 5(b) highlights that Q2 (corresponding to A2: willing to spend time and money improving health), Q18 (corresponding to B11: understand the side effects of diabetes-related drugs), Q25 (corresponding to C5: annual regular physical examination), and Q36 (corresponding to D7: can use traditional media such as paper literature and bulletin boards to obtain health education information) are identified as having relatively high importance for health awareness, knowledge, behavior, and skills, respectively.

## 4. Discussion

In the context of precision medicine and personalized healthcare, the level of HL among diabetic patients plays a significant and positive role in their self-health management [25, 26]. This study developed the DHLEIS, a HL evaluation index system specifically for diabetic patients, and designed a corresponding HL questionnaire. A survey was conducted among diabetic patients across five hospitals, utilizing RF and other analytical methods to thoroughly examine the impact of various influencing factors on overall HL and its four dimensions. Additionally, the core influencing factors were identified, and their contributions were analyzed to support precise interventions and personalized management of HL in diabetic patients.

From an overall and multidimensional perspective of HL, nine factors—age, sex, body type, income, exercise, education level, DM duration, insulin injection, and number of cohabitants—significantly impact HL level. Among these, age, DM duration, education level, and number of cohabitants show greater importance across the four dimensions of health knowledge, awareness, behavior, and skills. Several studies indicate that as the duration of the DM increases, HL and disease management experience tend to improve [2, 27]. However, the findings of this study are the opposite, indicating that HL levels decrease with the increased duration of the disease, aligning with the conclusions of Jafari et al. [9] and Mogessie et al. [28]. The reason for this outcome may include a lack of awareness regarding the long-term complications of DM [29], reduced sensitivity to the disease, or cognitive decline [30]. Other contributing factors may include low self-efficacy due to persistent lifestyle habits [31] (such as poor dietary management [32]) that are difficult to change or the lack of continuous support from health resources due to geographical factors [33], all of which can result in a decline in HL as the duration of DM increases.

There is a significant relationship between education level and both health knowledge and skills (positive correlation coefficients: 0.41 and 0.63, respectively), consistent with the findings of İlhan et al. and Finbråten et al. [34, 35]. Therefore, as illustrated in the characteristic portrait of the diabetic population (Figure 2), it is essential to design and implement targeted health promotion plans for populations with lower educational levels and older age. Studies from multiple regions indicate that interventions such as demonstration feedback [36], short message delivery interventions [37], and customized education supported by the physical environment [2] are more effective in improving HL for these groups, particularly in terms of user experience and feasibility.

The significance of secondary indicator factor Q30 (D1: understanding written medical orders and medication instructions) is remarkably substantial, reinforcing the assertion by Rafferty et al. that difficulties in understanding oral and written health information impede patients' active engagement in health management [38]. Currently, the correlation between cohabitation and HL remains inconsistent [39], with limited studies exploring the relationship between the number of cohabitants or family members and HL [40]. However, this study identifies a positive correlation between the number of cohabitants (family member count) and DM health skills, potentially attributable to support and assistance from family members, including younger children.

Both RF and PCA reveal that health knowledge and skills are crucial in influencing the level of HL, while the impact of health awareness and behavior is relatively minor. Unexpectedly, the significance of the other dimensions does not align consistently with the DHLEIS system based on expert opinions, except for health skills [41]. The hierarchy of importance presented in Table 2 corresponds with the theoretical comprehensive concept model of DHL proposed by Sørensen et al. [42] and van der Vaart and Drossaert [43]. This model posits that proficient health skills foster healthier behaviors and improved health outcomes. The disparities also indicate that in the relational concept of HL, individual-level factors and system demands interact and determine HL [44]. Furthermore, there is a specific sequence in this relational framework such as the level of HL influencing the degree to which health behaviors are implemented [45] rather than health behaviors dictating the level of HL (i.e., the relationship between HL and health efficacy [46]). Adequate health knowledge is recognized as an important factor influencing the evolution of patients' coping strategies, which suggests that the more patients understand their conditions, the better they can manage their health [47], a finding corroborated in this study. These insights provide direction for further research design and reference for the forthcoming theoretical update of DHLEIS.

### 4.1. Innovations and Limitations

The innovations of this study are manifold. Firstly, the study leverages expert opinion to construct a HL evaluation index system specifically for diabetic patients, providing a standard for the multidimensional comprehensive evaluation of HL. Secondly, it develops a standardized questionnaire based on this standard to comprehensively assess the HL of diabetic patients. Last but not least, the study utilizes ML and multidimensional deep analysis to examine the impact of different levels of influencing factors on HL, identifying key factors and thereby developing a rapid assessment tool for the HL of diabetic patients (patent pending). The identification of key factors not only improves communication efficiency between healthcare providers and patients and enhances patient compliance but also supports the formulation of clinical decision-making and optimizes the allocation of medical resources.

However, the representativeness and external validity of the study may be limited by the use of convenience sampling and the exclusion of participants with cognitive disabilities. Additionally, the limitations of the DHLEIS framework and the choice of ML methods may render the results somewhat deficient in contextual adaptation and generalization. Therefore, it is essential to further enhance and optimize the representativeness and generalization capability of the data in subsequent studies to enable wider applications in smart health management for DM.

## 5. Summary and Prospects

Based on the HL assessment standard system, analyzing the multidimensional factors influencing HL can help characterize the features of populations with varying levels of HL, enabling targeted cognitive improvements and effective enhancement of health skills. This provides a framework for subsequent studies on group differences and personalization of HL, offering clear direction for further adjustments to the general applicability of this study.

With continuous advancements and empowerment from technology, the evaluation and intervention of factors influencing diabetes HL will become increasingly straightforward and efficient. Additionally, considering regional and individual differences allows patients to achieve a higher level of health empowerment, enabling data-driven precise assessments, targeted improvements of HL levels, and meticulous self-health management.

## Figures and Tables

**Table 1:** Evaluation index system of health literacy of DM population and its corresponding weight.

**Primary indicators**	**Secondary indicators**
A. Health awareness (0.185)	A1. Believing that health is more important than money (0.025)
	A2. Willing to spend time and money improving health (0.031)
	A3. Willing to access health education information (0.033)
	A4. Willing to change one's unhealthy lifestyle habits (0.023)
	A5. Willing to receive professional guidance from doctors or nurses (0.010)
	A6. Willing to receive peer education (0.052)

B. Health knowledge (0.185)	B1. Understand the blood glucose control standards of diabetic patients (fasting blood glucose, blood glucose 2 h after meal, and glycosylated hemoglobin) (0.010)
	B2. Understand the main symptoms and complications of diabetes (0.021)
	B3. Master the basic nursing methods of diabetes (0.019)
	B4. Understand the significance of regular return visits to diabetes (0.021)
	B5. Understanding the treatment methods for hypoglycemia (0.010)
	B6. Understand the significance of diet control on the control of diabetes (0.010)
	B7. Understanding the meaning of food glycemic index (0.041)
	B8. Understand which diet is suitable for one's own health condition (0.029)
	B9. Understanding the importance of mental health (0.033)
	B10. Understanding the manifestations of psychological issues such as anxiety and depression (0.037)
	B11. Understand the side effects of diabetes-related drugs (0.027)
	B12. Mastering the correct use of hypoglycemic drugs (0.010)
	B13. Understand the relevant knowledge required to use a computer to access the internet (browser, website, and search tools) (0.039)
	B14. Learn about using smartphones to obtain health support (APP and WeChat official account) (0.035)

C. Health behavior (0.260)	C1. Nonsmoking (0.023)
	C2. Not drinking alcohol (0.033)
	C3. Ensure 7–8 h of sleep per day (0.033)
	C4. Reasonably arrange one's daily diet (0.010)
	C5. Annual regular physical examination (0.014)
	C6. Persist in using scientific and reasonable methods for physical exercise (0.014)
	C7. Exercise for at least half an hour every day (0.019)
	C8. Actively cooperate with doctors or nurses for examination (0.014)
	C9. Discover health issues and seek medical attention promptly (0.027)

D. Health skills (0.370)	D1. Can read written information such as medical orders and drug instructions (0.019)
	D2. Be able to use medication correctly according to medical advice (or inject insulin correctly) (0.014)
	D3. Be able to use a blood glucose monitor correctly (0.019)
	D4. Can use smart monitoring devices such as smartphones or wearable devices to monitor one's own health data (0.033)
	D5. Able to seek medical treatment online (online registration, doctor-patient online communication, etc.) (0.045)
	D6. Can use the internet to search for necessary health education information (0.033)
	D7. Can use traditional media such as paper literature and bulletin boards to obtain health education information (0.035)
	D8. Able to communicate with professionals to obtain health education information (0.040)
	D9. Can understand and judge the correctness of the health education information obtained (0.030)
	D10. Being able to make decisions that are beneficial for improving one's own health status based on one's own health status and educational information (0.029)

**Table 2:** Basic information of the survey subjects.

**Survey items**	**Subgroups**	**HL-adequate (** **n** = 387**)**	**HL-deficient (** **n** = 320**)**	**p** ** value**
Sex (%)	Male	251 (64.9)	159 (49.7)	< 0.001
	Female	136 (35.1)	161 (50.3)	

Age (%)	< 30	27 (7.0)	3 (0.9)	< 0.001
	30–49	104 (26.9)	30 (9.4)	
	50–79	240 (62.0)	252 (78.8)	
	80+	16 (4.1)	35 (10.9)	

DM duration (%)	< 5	167 (43.2)	116 (36.2)	0.039
	5–9	95 (24.5)	79 (24.7)	
	10–14	42 (10.9)	42 (13.1)	
	15–19	43 (11.1)	58 (18.1)	
	20+	40 (10.3)	25 (7.8)	

DM type (%)	T1DM	30 (7.8)	23 (7.2)	0.888
	T2DM	357 (92.2)	297 (92.8)	

Family history (%)	Yes	133 (34.4)	101 (31.6)	0.479
	No	254 (65.6)	219 (68.4)	

Body shape (%)	Standard	174 (45.0)	147 (45.9)	0.636
	Obesity	56 (14.5)	39 (12.2)	
	Overweight	108 (27.9)	99 (30.9)	
	Lean	49 (12.7)	35 (10.9)	

Marital status (%)	Single	14 (3.6)	10 (3.1)	0.1
	Married	368 (95.1)	298 (93.1)	
	Divorced/widowed	5 (1.3)	12 (3.8)	

Education level (%)	ILL	3 (0.8)	43 (13.4)	< 0.001
	PS	44 (11.4)	91 (28.4)	
	JHS	124 (32.0)	118 (36.9)	
	SEC	122 (31.5)	50 (15.6)	
	UG	91 (23.5)	17 (5.3)	
	MA+	3 (0.8)	1 (0.3)	

Income (%)	< 3000	28 (7.2)	42 (13.1)	< 0.001
	3000~5000	120 (31.0)	126 (39.4)	
	5000~10,000	120 (31.0)	62 (19.4)	
	> 10,000	87 (22.5)	52 (16.2)	
	Unclear	32 (8.3)	38 (11.9)	

Complication (%)	Yes	166 (42.9)	157 (49.1)	0.118
	No	221 (57.1)	163 (50.9)	

Exercise (%)	No	268 (69.3)	255 (79.7)	0.002
	Yes	119 (30.7)	65 (20.3)	

Dietary control (%)	No	113 (29.2)	104 (32.5)	0.387
	Yes	274 (70.8)	216 (67.5)	

OHA (%)	No	114 (29.5)	88 (27.5)	0.624
	Yes	273 (70.5)	232 (72.5)	

No intervention (%)	No	383 (99.0)	316 (98.8)	1
	Yes	4 (1.0)	4 (1.2)	

No. of cohabitant (%)	1	19 (4.9)	24 (7.5)	< 0.001
	2	183 (47.3)	179 (55.9)	
	3	114 (29.5)	36 (11.2)	
	4	25 (6.5)	30 (9.4)	
	5	35 (9.0)	27 (8.4)	
	6	11 (2.8)	21 (6.6)	
	7	0 (0.0)	3 (0.9)	

*Note:p* < 0.05, statistically significant.

Abbreviations: DM, diabetes mellitus; HL, health literacy; ILL, illiterate; JHS, junior high school; OHA, oral hypoglycemia agent; PS, primary school; SEC, high school/vocational school/technical secondary school; UG, college/undergraduate.

**Table 3:** The impact of four dimensions on health literacy level.

	**Health knowledge**	**Health skills**	**Health awareness**	**Health behavior**
MDI	124.43905	105.48082	51.83094	44.86886
PCA	0.79163	0.77622	0.65468	0.5239

Abbreviations: MDI, mean decrease in impurity; PCA, principal component analysis.

## Data Availability

The data that support the findings of this study are available on request from the corresponding author. The data are not publicly available due to privacy or ethical restrictions.

## Supporting Information

Supporting Information Additional supporting information can be found online in the Supporting Information section. Supporting Information S1. Original questionnaire on health literacy of people with diabetes.
