# Supplementary material for: Analysis of Influencing Factors Related to Health Literacy of Diabetic Patients: A Survey Based on DHLEIS
Source: J Diabetes Res. 2024 Nov 25;2024:5110867. doi: 10.1155/jdr/5110867 (PMC11611418; doi:10.1155/jdr/5110867)
Supplement: Supporting Information — Additional supporting information can be found online in the Supporting Information section. Supporting Information S1. Original questionnaire on health literacy of people with diabetes. [file 5110867.f1.doc]

**Supplementary Material 1**

**Questionnaire on health literacy of people with diabetes**

**Survey location:**_______________ **Survey date:** Year Month Day **Surveyor's signature:**__________  **Patient's hospital/clinic ID:**

Dear Friends:

Hello! In order to understand the health literacy level of the diabetic population, we sincerely invite you to participate in the survey and guarantee that all the information you provide will be strictly confidential. Please feel free to answer the questions truthfully. Thank you for your support!

Department of Medical Informatics, School of Medicine, Nantong University

January 2022

**Section One: Personal Basic Information**

**Note: Please fill in the blanks below or tick "√" in front of the corresponding options.**

**1.Your gender:** □ Male **□**female

**2.Your age this year: ______ years old**

**3.Your type of diabetes:**

□ Type I diabetes □ Type II diabetes □ Gestational diabetes □ Other

**4.You were diagnosed with diabetes ______ years ago.**

**5.Does your family have a history of diabetes?** □ Yes **□**no

**6.Your current body shape:**

**□**Thin **□**Standard **□**Overweight **□**Obesity

**7.Your education level:**

**□**Illiterate or barely literate **□**Primary school **□**Junior high school

**□**High school/vocational school/ Technical secondary school

**□**College/undergraduate **□**Master degree and above

**8.Your marital status:**

**□**Unmarried **□**Married **□**Divorced/Widowed **□**Other

**9.Your current or previous occupation:**

**□**Farmer **□**Teacher **□**Medical staff **□**Student

**□**Personnel from other public institutions **□**Farmers **□**Worker

**□**Other corporate personnel **□**Unemployed **□**Other

**10.Your family's average monthly income (yuan):**

**□**＜3000 **□**3000~5000 **□**5000~10000 **□**＞10000 **□**Not sure

**11. Current number of people living together: _____people**

**12.Do you have common complications of diabetes** (lesions of the heart, brain, kidneys, peripheral nerves, eyes, feet, etc.)

**□**Yes **□**No

**13.Your current diabetes treatment plan is** (multiple choices are allowed):

**□**none **□**Diet control **□**Physical exercise **□**Oral hypoglycemic drugs

**□**Injectable drugs □ Other (please specify)

**Section Two: Diabetes Health Literacy**

**Note: Please tick “√” in front of the options you think are appropriate based on your daily behavior and true feelings in life.**

**1. Health awareness**

**Q1.Health is more important than money. Do you agree?**

**□**Strongly disagree **□**Less agree **□**generally **□**More agree **□**Strongly agree

**Q2.Are you willing to invest time and money into improving your health?**

**□**Very unwilling **□**Less willing **□**generally **□**More willing **□**Very willing

**Q3.Are you willing to take the initiative to obtain health education information?**

**□**Very unwilling **□**Less willing **□**generally **□**More willing **□**Very willing

**Q4.Are you willing to change your bad living habits?**

**□**Very unwilling **□**Less willing **□**generally **□**More willing **□**Very willing

**Q5.Are you willing to accept professional guidance from a doctor or nurse?**

**□**Very unwilling **□**Less willing **□**generally **□**More willing **□**Very willing

**Q6.Peer education refers to bringing together people with diabetes who have similar backgrounds and experiences to share experiences, discuss problems, and provide support. Are you willing to receive such peer education?**

**□**Very unwilling **□**Less willing **□**generally **□**More willing **□**Very willing

**2. Health knowledge**

**Q7.The ideal fasting blood sugar control target for patients with type 2 diabetes is:**

**□**3.9~7.2mmol/L **□**8.0~10.0mmol/L **□**have no idea

**Q8.The ideal blood sugar control target for patients with type 2 diabetes is:**

**□**＜10.0mmol/L **□**＜12.0mmol/L **□**have no idea

**Q9.The classic symptoms of diabetes mainly include** (multiple choices are allowed):

**□**Drink more **□**Eat more **□**Polyuria **□**Weight loss in a short period of time

**Q10.Common complications of diabetes include** (multiple choices are allowed):

**□**Diabetic nephropathy **□**Retinopathy **□**Diabetic foot

**□**Neuropathy □Cardiovascular disease **□**Not sure

**Q11.How well do you understand the basic care methods for diabetes?**

**□**I don't understand at all **□**learn **□**familiar **□**skilled **□**proficient

**Q12.Regular follow-up visits for diabetes patients can help guide diabetic patients to make timely adjustments to their treatment plans, thereby achieving better individualized treatment and better controlling blood sugar. Do you agree with this statement:**

**□**Strongly disagree **□**Less agree **□**generally **□**More agree **□**Strongly agree

**Q13.If a diabetic patient has symptoms of hypoglycemia such as hand tremors, palpitations, and cold sweats, what do you think should be done (multiple choices are allowed):**

**□**Eating sugary foods **□**15Retest blood sugar after 15 minutes

**□**If the condition is serious, you should go to the hospital immediately **□**Not sure

**Q14.The significance of dietary control in controlling diabetes is (multiple choices are allowed):**

**□**Correction of dietary disturbances in glucose metabolism

**□**Maintain ideal weight

**□**Make blood sugar and blood lipids reach or approach normal levels

**□**Reduce the incidence of complications

**□**Not sure

**Q15.Which of the following foods are more likely to cause blood sugar to rise (multiple choices are allowed)?**

**□**Soba noodles **□**Fried dough sticks **□**Momordica charantia **□**Soda

**□**watermelon **□**Xylitol **□**granulated sugar **□**Not sure

**Q16.Do you think mental health is important for people with diabetes?**

**□**Not sure **□**unimportant **□**generally **□**important **□**Very important

**Q17.Do you understand the symptoms of psychological problems such as anxiety and depression?**

**□**Don't understand **□**Less known **□**Understand the general

**□**Learn more **□**Very understanding

**Q18.Do you know the side effects of diabetes-related medications?**

**□**Don't understand **□**Less known **□**Understand the general

**□**Learn more **□**Very understanding

**Q19.How much do you know about Baidu's information query function?**

**□**Don't understand **□**Less known **□**Understand the general

**□**Learn more **□**Very understanding

**Q20.How much do you know about health management applications (APPs) and hospital WeChat public accounts?**

**□**Don't understand **□**Less known **□**Understand the general

**□**Learn more **□**Very understanding

**3. Healthy Behavior**

**Q21.Do you smoke:**

**□**Regular smoking **□**Sometimes smoking **□**Occasional smoking

**□**Quit smoking **□**Never smoke

**Q22.Your drinking habits are:**

**□**Drinking alcohol frequently **□**Sometimes drinking **□**Occasionally drinking**□**Quit drinking **□**do not drink

**Q23.Do you have 7 ~ 8 Hours of sleep?**

**□**never **□**rare **□**sometimes **□**often **□**always

**Q24.Are you able to eat properly every day?**

**□**never **□**rare **□**sometimes **□**often **□**always

**Q25.How often do you have medical examinations?**

**□**Never check up **□**No pattern **□**1 time over 2 years

**□**Once a year **□**Once every six months

**Q26.Can you stick to scientific and reasonable exercise methods to do physical exercise?**

**□**very difficult **□**difficulty **□**generally **□**easy **□**very easy

**Q27.Do you exercise for at least half an hour every day?**

**□**never **□**rare **□**sometimes **□**often **□**always

**Q28.When you seek medical treatment, will you actively cooperate with the doctor or nurse's examination?**

**□**never **□**rare **□**sometimes **□**often **□**always

**Q29.When you find that you feel unwell or abnormal, will you seek medical attention in time?**

**□**never **□**rare **□**sometimes **□**often **□**always

**4. Health skills**

**Q30.Is it difficult for you to understand written information such as doctor's instructions and drug instructions?**

**□**very difficult **□**difficulty **□**generally **□**easy **□**very easy

**Diabetic patients who take medication should complete31(a)Patients who take insulin should complete31 (b)**

**Q31(a) Is it difficult for you to take your medicine on time every day?**

**□**very difficult **□**difficulty **□**generally **□**easy **□**very easy

**Q31 (b) Is it difficult for you to learn how to inject insulin correctly?**

**□**very difficult **□**difficulty **□**generally **□**easy **□**very easy

**Q32.Is it difficult for you to use a blood glucose monitor correctly?**

**□**very difficult **□**difficulty **□**generally **□**easy **□**very easy

**Q33.Is it difficult for you to monitor your own health data using smart monitoring devices such as smartphones or wearable devices?**

**□**very difficult **□**difficulty **□**generally **□**easy **□**very easy

**Q34.Is it difficult for you to use your smartphone to make online appointments or communicate with doctors online?**

**□**very difficult **□**difficulty **□**generally **□**easy **□**very easy

**Q35.Is it difficult for you to get the health education information you need from the Internet?**

**□**very difficult **□**difficulty **□**generally **□**easy **□**very easy

**Q36.Is it difficult for you to find the health education information you need from traditional media such as newspapers, blackboards, and flyers?**

**□**very difficult **□**difficulty **□**generally **□**easy **□**very easy

**Q37.Is it difficult for you to get health education information when communicating with diabetes professionals?**

**□**very difficult **□**difficulty **□**generally **□**easy **□**very easy

**Q38.Is it difficult for you to judge whether the health education information you receive is correct?**

**□**very difficult **□**difficulty **□**generally **□**easy **□**very easy

**Q39.Is it difficult for you to make decisions based on your health and education information to improve your health?**

**□**very difficult **□**difficulty **□**generally **□**easy **□**very easy

**The survey ends here, thank you for your cooperation!**
